# Draft Genome Sequence of *Pseudoruegeria* sp. SK021, a Representative of the Marine *Roseobacter* Group, Isolated from North Sea Sediment

**DOI:** 10.1128/genomeA.00541-17

**Published:** 2017-06-15

**Authors:** Marion Pohlner, Ian Marshall, Lars Schreiber, Heribert Cypionka, Bert Engelen

**Affiliations:** a Institute for Chemistry and Biology of the Marine Environment, University of Oldenburg, Oldenburg, Germany; b Department of Bioscience, Center for Geomicrobiology, Aarhus University, Aarhus, Denmark

## Abstract

*Pseudoruegeria* sp. SK021 is a member of the *Roseobacter* group, isolated under aerobic conditions from North Sea sediment. The draft genome comprises 3.95 Mb and contains 3,747 protein-coding sequences. Although the strain is nonmotile under laboratory conditions, the entire set of genes for the formation of a flagellar apparatus was found.

## GENOME ANNOUNCEMENT

The *Roseobacter* group is globally distributed in the marine environment and represents a significant part of pelagic and benthic microbial communities (1–3). Their broad metabolic versatility make roseobacters successful in a variety of habitats (4, 5). In coastal sediments, roseobacters can constitute up to 10% of all cells (6). Although 28% of all described species in this group are of benthic origin (7), the metabolic properties of roseobacters in sediments are poorly understood. *Pseudoruegeria* sp. SK021, analyzed in this study, was isolated from surface sediment of the North Sea (7.1667 E, 57.8145 N) at a water depth of 181 m below sea level (8). It is closely related to *P. aestuarii* and represents a new benthic member of this genus.

*Pseudoruegeria* sp. SK021 was grown on marine broth agar (Difco) amended with dimethyl sulfide (100 µM) and lactate (5 mM) at 20°C. DNA was extracted using the innuPREP DNA mini kit (Analytik Jena) and a sequencing library was prepared using the Nextera XT kit (Illumina). Genome sequencing was performed using the Illumina MiSeq platform with the MiSeq reagent kit version 3 and generated approximately 3.8 million reads, representing ~0.98 Gb of data (fastq-stats version 1.01, http://expressionanalysis.github.io/ea-utils). Reads were trimmed and adapters removed using Trimmomatic version 0.36 (9) with the following parameters: CROP:288, HEADCROP:19, SLIDINGWINDOW:4:20, MINLEN:100, ILLUMINACLIP:bbmap/adapters.fa:2:40:15. Paired reads were assembled with SPAdes version 3.9.1 (10) using “--careful” and multiple *k*-mer sizes (-*k* 21, 33, 55, 77, 99, 127). Only contigs with a G+C content of 40 to 68%, an average read coverage > 7.5×, and a minimum size of 200 bp were retained to eliminate potential contamination. After decontamination, the assembled draft genome of *Pseudoruegeria* sp. SK021 had a total length of 3,948,746 bp, 128 contigs (>500 bp), and approximately 245-fold coverage. The average G+C content was 60.17% and the *N*_50_ length was 94,596 bp, as determined by QUAST version 4.3 (11). Genome completeness was 99.27%, estimated by CheckM version 1.0.7 (12) using marker genes for the family *Rhodobacteraceae*. Annotation by Prokka version 1.12-beta (13), on the basis of three published and annotated genomes of *Pseudoruegeria* spp., identified 3,747 protein-coding sequences, 3 rRNA-encoding sequences (5S, 16S, 23S rRNA), and 48 tRNAs. Even though *Pseudorugeria* sp. SK021 is nonmotile, genes for the formation of the complete flagellar apparatus were found in the annotated genome, including genes for the motor switch (e.g., FliG, FliM, MotA), the basal body (e.g., FlgB, FlgC), the different rings (FliF, FlgH, FlgI), the flagellar hook (e.g., FlgE, FlgK, FlgL), and the flagella itself (flagellin). Although motility is not essential in sediments, the presence of flagellar genes shows that the strain might be motile under specific conditions.

### Accession number(s).

The genome was uploaded to IMG under Genome ID 2711768631. Furthermore, this whole-genome shotgun project has been deposited at DDBJ/ENA/GenBank under the accession number MTBG00000000. The version described in this paper is the first version, MTBG01000000.
